# Structure and transformation of tactoids in cellulose nanocrystal suspensions

**DOI:** 10.1038/ncomms11515

**Published:** 2016-05-04

**Authors:** Pei-Xi Wang, Wadood Y. Hamad, Mark J. MacLachlan

**Affiliations:** 1 Department of Chemistry, University of British Columbia, 2036 Main Mall, Vancouver, British Columbia, Canada V6T 1Z1; 2 FPInnovations, 2665 East Mall, Vancouver, British Columbia, Canada V6T 1Z4

## Abstract

Cellulose nanocrystals obtained from natural sources are of great interest for many applications. In water, cellulose nanocrystals form a liquid crystalline phase whose hierarchical structure is retained in solid films after drying. Although tactoids, one of the most primitive components of liquid crystals, are thought to have a significant role in the evolution of this phase, they have evaded structural study of their internal organization. Here we report the capture of cellulose nanocrystal tactoids in a polymer matrix. This method allows us to visualize, for the first time, the arrangement of cellulose nanocrystals within individual tactoids by electron microscopy. Furthermore, we can follow the structural evolution of the liquid crystalline phase from tactoids to iridescent-layered films. Our insights into the early nucleation events of cellulose nanocrystals give important information about the growth of cholesteric liquid crystalline phases, especially for cellulose nanocrystals, and are crucial for preparing photonics-quality films.

## Abstract (web summary)

Cellulose nanocrystals suspensions self-assemble into cholesteric liquid crystalline droplets, namely tactoids, which then condense into helical structures upon drying. Here, Wang *et al*. capture the structural evolution of this nucleation process by trapping and protecting tactoids in a polymer matrix.

## Graphical abstract

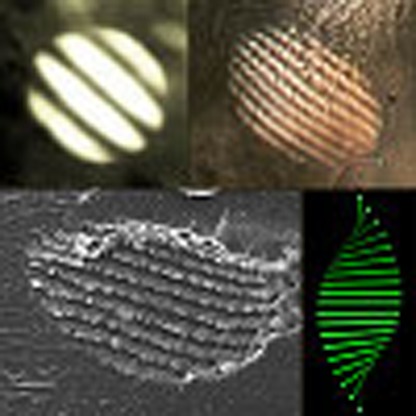

Cellulose nanocrystals (CNCs) have impressive structural and mechanical properties that are being exploited for diverse applications^1^,^2^,^3^,^4^,^5^. In 1959, Marchessault *et al*.^6^ reported the formation of permanently birefringent gels from colloidal dispersions of cellulose crystallites after acid hydrolysis. Interestingly, liquid crystalline droplets (called ‘tactoids') spontaneously nucleate from aqueous CNC suspensions above ∼3 wt% CNCs^7^. Drying the suspensions affords iridescent films^8^,^9^ with a helicoidal-layered structure resembling the Bouligand structure of chitin observed in crabs, beetles and other arthropods^10^,^11^. As the reflected colour of the films can be changed by varying the pitch of the helical structure^12^,^13^, these iridescent materials are of great interest for coatings^14^, security features^15^, and sensors^16^.

Despite growing interest in the solution and solid-state properties of CNCs, there is still a large gap in our understanding of the liquid crystallinity and structure of these materials. One hypothesis is that the chirality arises from a helical twist in the crystals^17^,^18^, and several researchers have observed twisting of cellulose microfibrils^19^,^20^ and CNCs^21^ by electron microscopy and atomic force microscopy, but the twisting is quite subtle. Bergström and coworkers^22^ showed that the chiral nematic phase is already established when the CNCs are ∼50 nm apart in water. It is not clear how chirality is mediated between the CNCs over these distances. Some researchers have postulated that there is a chiral charge distribution of the sulfate ester groups on the surface emanating from the H_2_SO_4_ hydrolysis process^7^. The transformation from isotropic to fully lyotropic liquid crystal in the case of CNCs has been difficult to study, but critical understanding may come from the structure of tactoids, which are key components in the evolution of liquid crystallinity in CNCs.

Tactoids are spherical, ellipsoidal or spindle-shaped anisotropic droplets nucleated with sharp edges in isotropic dispersions^23^. They have been observed in many liquid crystalline substances, including polypeptides^24^,^25^. As these tactoids with short-range-ordered structures are the intermediate state that bridges the isotropic phase and the macroscopic liquid crystalline phase with long-range anisotropy^26^,^27^, great efforts have been made in the past 50 years to explain their origin, shape and structure; alas, these studies were nearly all based only on polarized optical microscopy (POM) or X-ray diffraction^28^,^29^,^30^. The significance of tactoids in improving the properties of liquid crystal-derived materials has been noticed, since Lagerwall and coworkers^31^ showed that applying shear forces to suspensions of CNCs as they dried improved the alignment of the tactoids and, consequently, improved the optical properties of the films. While hypothetical models have been built, the structure of these tactoids and the arrangement of the dispersed substances within them have not been directly observed with electron microscopy due to the difficulty of capturing these droplets in a solid-state matrix while maintaining their ordered structures.

Here we demonstrate a way to capture CNC tactoids from suspensions and solidify them in polymer matrices as ‘fossil records'. This new method enables us to directly observe, for the first time, the arrangement of CNCs inside the tactoids by electron microscopy, and study the morphological transformation process from distinct tactoids to long-range lamellar structures with strong iridescence.

## Results

### Solidification of CNC tactoids in PAAm matrices

When the lyotropic liquid crystal of CNCs first forms, one can observe tactoids by POM under crossed polarizers (Supplementary Movie 1). These tactoids appear as anisotropic droplets with parallel birefringent bands that are ∼10 μm apart. Newborn tactoids (those having the fewest bands) usually have an ellipsoidal shape, possibly due to the interfacial tension between the isotropic and anisotropic phases^32^. The spacing between adjacent periodic bands is much longer (more than 10 times) than the length of the CNCs, which is consistent with a chiral nematic structure rather than smectic order, as in smectic phases the spacing of periodic bands should correspond to the length of the mesogens^7^,^33^. Our initial efforts to capture these tactoids by rapidly freezing the suspensions and then freeze-drying failed to show anything sensible due to collapse of the structure when water is removed. We postulated that capturing the tactoid in a polymer matrix may prevent the collapse. As it was previously demonstrated that the precursors of polyacrylamide (PAAm) hydrogels are compatible with the self-assembly of CNCs in aqueous suspensions^16^, we wondered if a similar polymerization process could be used to capture the microscopic structure and transformation of tactoids.

**Figure 1:**
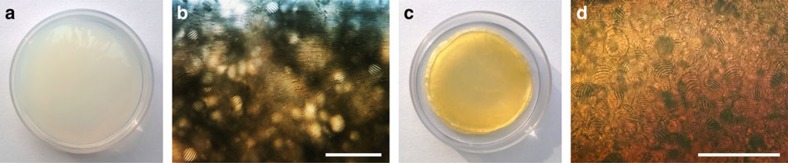
Photos and POM images of polymer matrices with CNC tactoids. (**a**) Freshly prepared CNC–PAAm composite hydrogel. (**b**) POM image of a fresh hydrogel section. (**c**) Dried gel. (**d**) POM image of a dried hydrogel section. Evaporation time and estimated CNC concentration for these images are 6 h and 4.4 wt%. Diameter of the Petri dishes in **a** and **c** is 60 mm. Scale bars, 200 μm (**b** and **d**).

Suspensions of CNCs in water were combined with PAAm precursors and a photoinitiator. After sonication, the mixtures were allowed to stand in 60 mm Petri dishes for a period (‘evaporation time') of 1–12 h under ambient conditions. The mass of these CNC–PAAm precursor mixtures decreases linearly with time, as shown in Supplementary Table 1, causing the concentration of CNCs to vary from ∼4 to 5 wt% CNCs over this time. After a period of evaporation, tactoids were clearly visible by POM and photopolymerization was initiated with UV light. Figure 1a shows a photograph of the freshly prepared hydrogel that was polymerized after an evaporation time of 6 h (∼4.4 wt% CNCs), and Fig. 1b shows the POM image of a cross-section of this hydrogel, clearly revealing tactoids with periodically spaced birefringent bands. After solvent removal, the hydrogels turned into hard and brittle plastic blocks (Fig. 1c) with the microstructures of the tactoids stabilized inside the matrix (Fig. 1d). Thus a ‘fossil record' that represents a transition stage of the CNC liquid crystalline phase morphological transformation was obtained, giving us an unprecedented chance to reveal the microscopic origins of tactoids, fingerprints and other optical phenomena of CNC suspensions by electron microscopy.

### Chiral nematic organization of CNCs in tactoids

**Figure 2:**
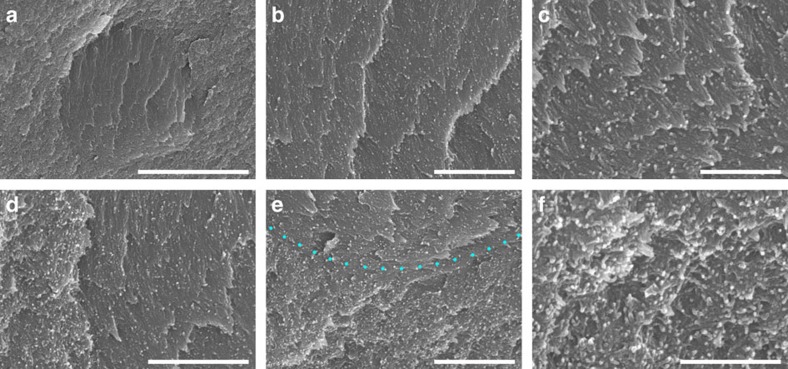
SEM images of a baby tactoid captured in a polymer matrix. (**a**) Cross-sectional SEM image of a newly formed CNC tactoid (1 h evaporation time, about 4.1 wt% CNCs). The ordered arrangement of CNCs inside this tactoid is shown in **b** and **c**. (**d**) The left edge of this tactoid. (**e**) The bottom edge of the same tactoid. (**f**) The randomly arranged CNCs in the nearby isotropic region outside this tactoid. Scale bars, (**a**) 5 μm, (**b**) 1 μm, (**c**) 500 nm, (**d**,**e**) 1 μm and (**f**) 500 nm.

Scanning electron microscopy (SEM) of dried hydrogel cross sections revealed tactoids of various sizes. Figure 2a shows the SEM image of a ‘baby' tactoid formed, a tiny droplet without birefringent bands, as verified by POM (Supplementary Fig. 1). SEM clearly reveals that these ‘baby' tactoids are ellipsoidal, anisotropic microdomains with a lamellar-like structure. CNCs in these ‘baby' tactoids are arranged parallel into thin nematic layers, as shown in Fig. 2b,c. No periodic bands (that is, birefringent lines) could be observed in these tiny tactoids as their diameters are <5 μm, which is not enough to contain a half-helical pitch of the CNC chiral nematic structure at that stage (usually in the range of 5–10 μm). Although individual CNC rods can been seen, their helical twisting could not be resolved at this stage.

**Figure 3:**
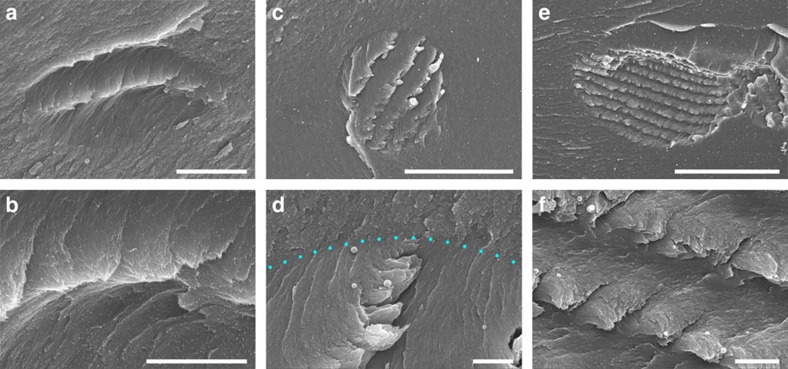
SEM images of tactoids with different numbers of periodic bands. (**a**) Cross-sectional SEM image of a tactoid with only one band. This band is magnified in **b**. (**c**) A tactoid with four periodic bands. Its top edge is indicated in **d** by blue dots. (**e**) A tactoid with nine periodic bands. A half helical pitch of this tactoid is shown in **f**. Evaporation time and estimated CNC concentration for these images are as follows: (**a**,**b**) 1 h, 4.1 wt%; (**c**,**d**) 3 h, 4.2 wt%; and (**e**,**f**) 6 h, 4.4 wt%. Scale bars, (**a**) 5 μm, (**b**) 2 μm, (**c**) 30 μm, (**d**) 2 μm, (**e**) 40 μm and (**f**) 2 μm.

As a liquid crystalline domain, the tactoid is separated from the isotropic region (where CNCs show no preferential alignment) by an arc-shaped sharp edge (Fig. 2d,e and also Fig. 3d). CNCs inside the tactoid are arranged in nematic layers (Fig. 2c), while outside this domain they are substantially more disordered, consistent with an isotropic phase (Fig. 2f). Additional images showing the details of this tactoid can be found in Supplementary Figs 2 and 3. The diameter of the nanocrystals in these tactoids is about 20–30 nm, while the CNCs used in these studies have widths of ∼10–30 nm and lengths of ∼100–400 nm (Supplementary Fig. 4). This difference could be caused by the polymer matrix, sputter coating (8 nm of 80/20 Pt–Pd alloy) or the possible aggregation of CNCs.

Tactoids with a diameter of 10–15 μm usually have only one periodic band, as shown in Fig. 3a, which was captured after an evaporation time of 1 h (∼4.1 wt% CNCs). Here it should be noticed that the tactoid looks flattened in the vertical direction, possibly due to the anisotropic shrinkage of the hydrogel when it was being dried (*vide infra*). The first periodic band in the newly formed tactoid looks like a wave crest (Fig. 3b). At this early stage, CNCs in a tactoid are twisted about 180° from one end to the other, which means a half-helical pitch. Another tactoid with only one band is shown in Supplementary Fig. 5.

After further water evaporation, larger tactoids with more birefringent bands are generated. Figure 3c shows a 30-micron-sized tactoid with four periodic bands. An unexpected aggregation of the chiral nematic layers into thick sheets is observed as a stepped geometry; a cross-section of such a sheet is shown in Supplementary Fig. 6a, which contains a half-helical pitch. This phenomenon may be caused by shear forces during the drying process of the hydrogels, cleaving the layers at a particular orientation of the CNCs in the layered structure. More SEM images pertaining to this phenomenon can be found in Supplementary Figs 6 and 7.

Figure 3e shows a tactoid with nine periodic bands, where CNCs are well organized into a chiral nematic structure with a periodic spacing of about 5 μm (Fig. 3f and Supplementary Fig. 8). We even observed individual tactoids with up to ∼30 bands forming in suspensions after a long evaporation time of 12 h (∼4.9 wt% CNCs). Supporting SEM images showing different growth stages of tactoids can be found in Supplementary Fig. 9.

**Figure 4:**
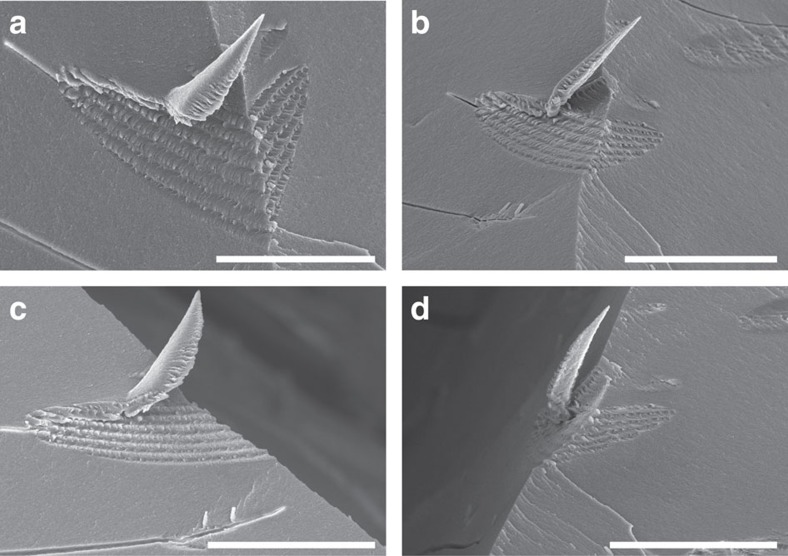
SEM images of a tactoid sitting at a right-angled edge. This tactoid has seven periodic bands (6 h evaporation time, about 4.4 wt% CNCs). Its top, front, left and right views are shown in **a**,**b**,**c** and **d**, respectively. Scale bars, (**a**) 40 μm and (**b**–**d**) 50 μm.

**Figure 5:**
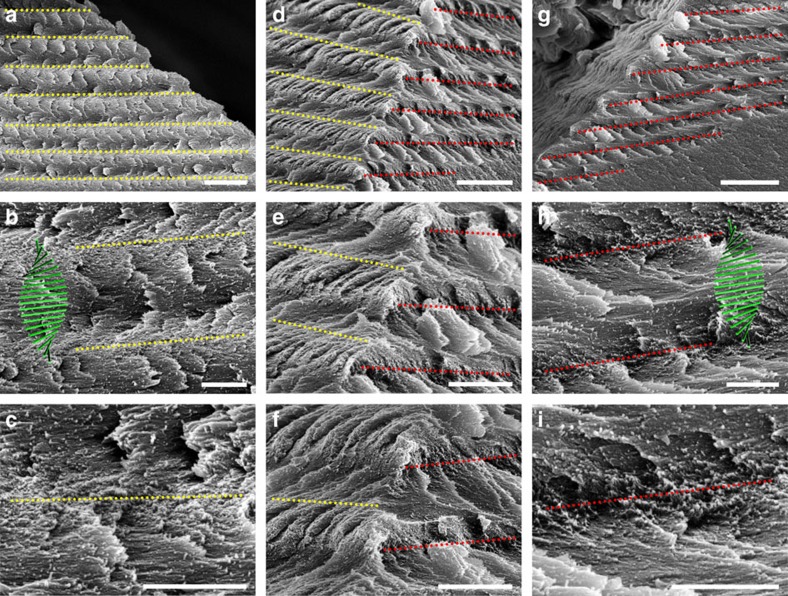
The three dimensional spatial structure of a tactoid. The tactoid shown in Fig. 4 is examined by SEM from different directions. Left, front and right views are placed in the left, middle, and right columns, respectively. On the left side, CNCs perpendicular to the cross-section (the CNCs projecting out) are indicated by yellow lines (**c**), while at the right side they are indicated by red lines (**i**). The region between two adjacent lines is a half helical pitch as shown in **b** and **h**, while the long-range spacing is depicted in **a** and **g**. From **d**–**f** we can see that the CNCs projecting out on the left and right sides of the tactoid are alternately arranged, which is a solid confirmation of the chiral nematic ordering in CNC tactoids. Scale bars, (**a**) 5 μm, (**b**,**c**) 1 μm, (**d**) 5 μm, (**e**,**f**) 2 μm, (**g**) 5 μm and (**h**,**i**) 1 μm.

We were fortunate to successfully capture tactoids at the intersection of two fracture surfaces, which allowed us the opportunity to view them from multiple angles. Figure 4 shows a tactoid with a right-angled edge (6 h evaporation time, ∼4.4 wt% CNCs), which was imaged from both the left (Fig. 5a–c), the front (Fig. 5d–f) and the right (Fig. 5g–i) sides. CNCs projecting outward perpendicular to the left side cross-section surface are clearly shown in Fig. 5c, which are indicated by a yellow dotted line. CNCs projecting out on the right side of the tactoid are indicated by a red dotted line (Fig. 5i). The region between two adjacent lines represents a half-helical pitch as shown in Fig. 5b,h, while the long-range spacing of these lines is depicted in Fig. 5a (left side) and Fig. 5 g (right side). By comparing all of these images, we are able to locate both the left- and right-oriented CNCs as shown by the yellow and red lines, respectively, in the front viewings of Fig. 5d–f. The relationship between the CNC orientations on the two sides is strong evidence for the chiral nematic ordering in the CNC tactoids. Additional SEM images of tactoids with a right-angled edge are shown in Supplementary Fig. 10.

Although the chiral nematic ordering of CNC tactoids was successfully captured by the PAAm hydrogels, the shrinkage of these hydrogels on drying inevitably decreased the size of the tactoids inside, and sometimes flattened them in the vertical direction. We found that a typical hydrogel that evaporated for 6 h before photopolymerization (that is, 4.4 wt% CNCs) shrank ∼66% in thickness (from 3.53±0.03 to 1.20±0.03 mm), and ∼43% in diameter (from 60 to 34±1 mm) after complete drying. If the hydrogel is polymerized with a lower volume fraction of water, it shrinks less on drying. For example, a hydrogel prepared after an evaporation time of 12 h shrank ∼61% in thickness (from 3.20±0.03 to 1.24±0.01 mm), and ∼43% in diameter. Thus the anisotropic shrinkage of hydrogels would vertically flatten the tactoids inside. In addition, the cross sections of tactoids are not always parallel to their helical axes, prohibiting accurate measurements of the helical pitch based on SEM images. This problem has been thoroughly discussed in the situation of chiral nematic CNC films by Majoinen *et al*.^34^ Nevertheless, we measured the pitch of the chiral nematic structure in 29 tactoids as accurately as possible (Supplementary Table 2). The pitch ranges from 4 to 12 μm, consistent with the ranges observed by POM. If the lateral growth of layers in a tactoid is faster than the generation of new layers, its helical axis would be directed along the short axis of the ellipsoid; otherwise it would be parallel to the long axis. Both of these two cases have been observed during the drying process of CNC suspensions (Supplementary Movie 1).

### Fusion of tactoids and formation of defects

**Figure 6:**
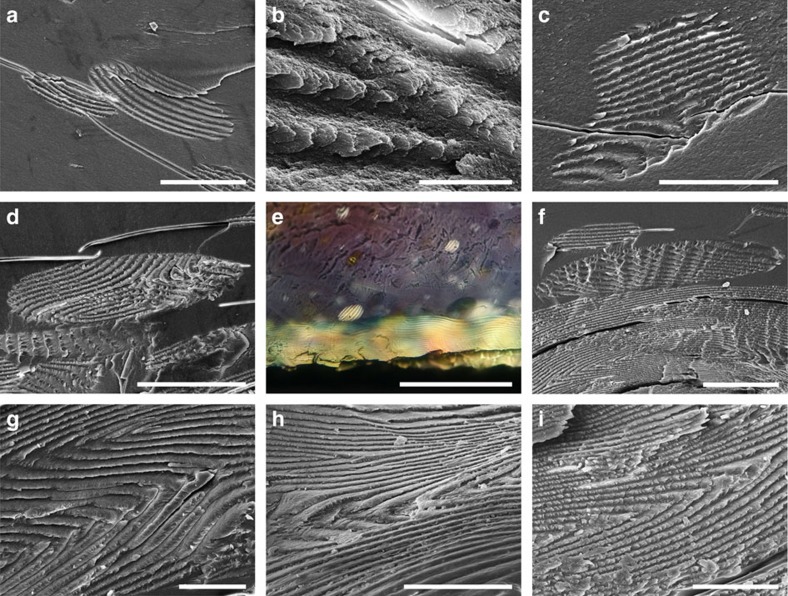
Fusion of tactoids and formation of defects. (**a**) SEM image depicting the initiation of fusion between two tactoids. (**b**) SEM image of the fusion point shown in **a** at a higher magnification. (**c**,**d**) ‘Hybrid tactoids' formed by the fusion of several individual ones. (**e**,**f**) POM and SEM images illustrating the sedimentation of CNC tactoids to the lower part and the formation of continuous layers at the bottom of the suspension. Tactoid fusion defects that persist in continuous layers are shown in SEM images (**g**,**h** and **i**). Evaporation time and estimated CNC concentration for these images are as follows: (**a**,**b**) 9 h, 4.6 wt%; (**c**–**i**) 12 h, 4.9 wt%. Scale bars, (**a**) 50 μm, (**b**) 5 μm, (**c**,**d**) 50 μm, (**e**) 200 μm, (**f**) 50 μm and (**g**–**i**) 20 μm.

As the concentration of CNC suspensions is increased, bigger tactoids with more periodic bands appear. Watching the tactoids in a drying suspension by POM for hours, we observed that in many situations the tactoids can grow by a coalescence mechanism, where smaller tactoids fuse to form larger tactoids (Supplementary Movies 2–5). This is a similar mechanism to the growth of oil droplets in emulsions. We were fortunate to capture these fusions in the PAAm hydrogels. Figure 6a shows a collision between two tactoids and Fig. 6b shows the orientations of the periodic bands of these tactoids at the contact point. We cannot preclude the possibility that other growth mechanisms, such as Ostwald ripening, are simultaneously active.

In some cases (especially when two tactoids come together with a right angle between their helical axes), the fusion between the tactoids leads to the formation of defects as their periodic bands bend, fold, elongate or dislocate in the process. Interestingly, the layers are soft and flexible enough to deform while accommodating the union, but often the two tactoids never completely fuse into a single uniform tactoid, possibly due to the slow relaxation rate of CNCs as their size is much larger than molecular cholesteric liquid crystalline substances. It was previously noted that healing of the defects into a uniform domain must take a long time under ambient conditions, particularly when evaporation is continuing^31^. Two hybrid tactoids formed by fusion processes are shown in Fig. 6c,d, where newly formed ones usually have irregular shapes (Fig. 6c), but gradually appear to become more regular ellipsoids (Fig. 6d, Supplementary Movie 4).

### Sedimentation of tactoids

We hypothesized that CNCs would be more closely packed inside tactoids as they adopt a similar orientation in each layer, enabling them to occupy the space more efficiently. This would lead to a higher density of CNCs inside the tactoids than in the isotropic regions. Quantifying the density of the tactoids has proven to be difficult since they are predominantly water. However, we could clearly observe aggregation of tactoids in the lower regions of the hydrogel (Supplementary Fig. 12d), and even observe sedimentation of birefringent layers at the bottom of the suspension from vertically sliced cross sections of fresh hydrogels, as shown in Fig. 6e,f. This phenomenon is consistent with the isotropic and ordered phase separation observed in CNC suspensions^26^,^35^. In our work, a suspension of 4.08 wt% CNCs was allowed to stand in a sealed separatory funnel for 1 week to equilibrate, and the concentration of the CNCs in the ordered phase was measured to be 4.46 wt%, while the CNC concentration in the isotropic phase was 3.75 wt% (densities of 1.0165 and 1.0150, g cm^−3^, respectively). Now it is apparent that the so called ‘ordered phase' in CNC suspensions has a long-range lamellar structure composed of fused tactoids, while discrete tactoids still exist in the upper phase. Fusions also happen between tactoids and continuous layers when discrete tactoids reach the bottom of the suspension and join the long-range lamellar structure, leading to the formation of fusion defects. More images showing the fusions of CNC tactoids can be found in Supplementary Fig. 11.

Eventually, the evaporation of water from the CNC suspension will press and flatten all the microstructures inside. The periodic bands will be mostly horizontally aligned and tightly packed together with the spacing among them being greatly decreased in comparison to the tactoids. However, the fusion defects of tactoids will persist in the continuous lamellar structure as shown in Fig. 6g–i. These defects generally have a much longer spacing (usually >10 μm) compared with other regions. We believe that these fusion defects are one of the possible origins of the fingerprint textures observed from the top of dried CNC films (pure or composite) with POM (Supplementary Fig. 12f). Recently Gray and Mu^36^ provided another explanation for the fingerprint textures based on the POM observation of concentrated (6–8 wt%) CNC suspensions. The predominant part of these systems is the liquid crystalline phase, and the long-range lamellar structures are already formed throughout. Flow of the suspension causes distortions and gives rise to an oblique viewing angle for the chiral nematic bands, which increases the apparent spacing of the fingerprint lines. But drying these concentrated suspensions can also lead to the formation of folded layers, and the periodic spacing of the vertexes of these folded layers will eventually be much larger than the half helical pitch.

## Discussion

**Figure 7:**
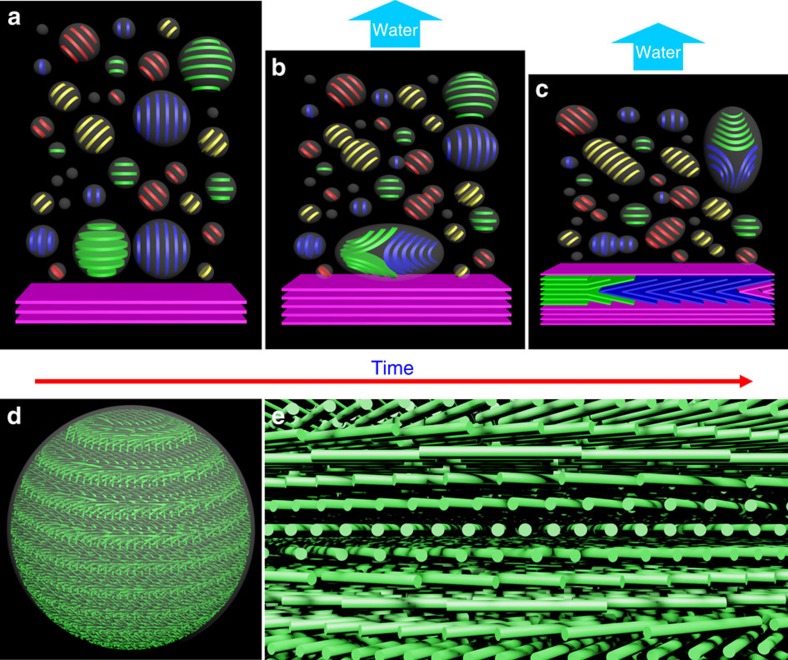
Models of the structure and transformation of tactoids. (**a**–**c**) Cartoon depictions of the tactoids and their transformations as the solvent evaporates from the CNC suspension. A typical fusion mode which leads to the defects of folded layers is shown. **d** and **e** are three-dimensional models showing the ellipsoidal shape of CNC tactoids and the chiral nematic ordering of CNCs inside them.

Our electron microscopy results show that the tactoids themselves have chiral nematic structure and they are already well organized. When tactoids grow by a coalescence mechanism (which is one possibility for the growth of tactoids; other mechanisms may also occur), the periodic bands of a large tactoid are gathered by fusions of small ones (Supplementary Fig. 13). Thus, each band represents a baby tactoid at the very early state, resulting in defects between every two adjacent bands. Although CNCs are reoriented to minimize these defects, they still undermine the continuity of the chiral nematic ordering in the whole tactoid. When these organized tactoids fuse and sediment, they form the layered, iridescent chiral nematic structure that is familiar for films of CNCs. Figure 7a–c are cartoon depictions of the tactoids and their transformations as the solvent evaporates, yielding long-range chiral nematic layers of CNCs. Two simplified situations for the fusion of tactoids are depicted. In one case, two tactoids come together with their helical axes perpendicular to each other (a ‘head-to-side' mode), afterwards a Y-shaped fusion defect is formed, as the bands of one tactoid are folded and inserted between two adjacent bands of the other tactoid. In this case, the fusion defect would be eventually retained in the continuous lamellar structure and then the dried CNC film. In another case, two tactoids fuse with their helical axes parallel to each other (a ‘head-to-tail' mode), thus a larger tactoid with more periodic bands is formed, and ideally no bands would be folded in this process. It should be noted that tactoids gradually flatten from spheres into oblate spheroids or ellipsoids during this process, while the orientation of the helical axis can change among tactoids and is not always aligned along the tactoid's short or long axis. Figure 7d depicts the chiral nematic arrangement of idealized CNCs in a typical tactoid; Fig. 7e is a magnified image of the helicoidal organization of the rods within the tactoid.

In summary, we report an effective method to capture the tactoids formed in aqueous suspensions of CNCs, and stabilize them in solid matrices. This method provides us with an unprecedented opportunity to directly observe the organization of CNCs in the tactoids at the nanoscale using electron microscopy. These studies reveal that the tactoids formed from the isotropic phase have chiral nematic order with a hierarchical-layered structure. Also, the morphological transformation from tactoids to long-range-ordered structures is studied, and the fusion defects of tactoids are observed. These defects become part of the fingerprint texture in dried CNC films. We hope that these results will help to guide the construction of CNC-based materials with improved order, and that our methods may be applied to understand the arrangements of mesogens in other liquid crystalline substances.

## Methods

### Capture of tactoids in polyacrylamide hydrogels

In a typical procedure, 1.0 g acrylamide (monomer), 100 mg N,N′-methylenebisacrylamide (cross-linker) and 5 mg 2-hydroxy-4′-(2-hydroxyethoxy)-2-methylpropiophenone (or 50 mg 2,2-diethoxyacetophenone, photoinitiator) were mixed with 10 ml CNC suspension (about 400 mg CNCs). After sonication for 1 h, the homogeneous mixture (total volume ∼11.2 ml) was poured into a 60-mm polystyrene Petri dish to evaporate until a desired microstructure was formed, which could be monitored in real-time by POM. This delay time is termed the ‘evaporation time' in the manuscript. A 300-nm ultraviolet-B light source (8 W) was used in the photopolymerization process. Ultraviolet irradiation was applied for 3 h to give CNC–PAAm composite hydrogels (in general, robust hydrogels could be obtained in 20 min).

In the morphological transformation experiments, a series of CNC–PAAm precursor suspensions was prepared as above and dried under ambient conditions in parallel. Ultraviolet light was applied to different batches of these samples after 1, 3, 6, 9, 12 and 24 h to capture the microstructures at different evaporation stages.

### Measurements

The cross sections used for POM imaging were vertically sliced from freshly prepared hydrogels with a razor blade, and then horizontally placed on glass slides for imaging. Regions at different depths in the suspensions could be observed when the cross sections were moved horizontally on the POM stage.

Samples for SEM imaging were obtained by heating the hydrogels in air at 60 °C for 12 h, then breaking the resulting hard and brittle plastic blocks into small pieces with a hammer.

In the phase separation experiment, a CNC suspension (∼200 ml) was placed in a sealed separatory funnel to stand for 7 days at 8 °C. The two phases were then separated and CNC concentrations were determined as follows: a 10 ml CNC suspension in a pre-weighed vial was weighed using an analytical balance, which was then completely dried at 75 °C for 24 h, cooled and weighed again. Densities were determined by measuring the mass of a suspension in a 10-ml volumetric flask and comparing with the mass of deionized water of the same volume.

## Additional information

**How to cite this article:** Wang, P.-X. *et al*. Structure and transformation of tactoids in cellulose nanocrystal suspensions. *Nat. Commun.* 7:11515 doi: 10.1038/ncomms11515 (2016).

## Supplementary Material

Supplementary Information Supplementary Figures 1-13, Supplementary Tables 1-2, Supplementary Methods and Supplementary Reference

Supplementary Movie 1 Polarized optical microscopy of a droplet of 4 wt% CNCs on a glass slide as the water evaporates. The movie (image area, 430x570 μm) was filmed with crossed polarizers over about 1 hour and has been sped up 60x. The microscope was refocused every 10 seconds during the experiment. Between 50 and 60 seconds, the sample fully solidifies into an iridescent film with defects from the merged tactoids apparent. After 1:10, the analyzer was rotated by 360° to show the birefringence.

Supplementary Movie 2 Polarized optical microscope image of two tactoids fusing along their helical axes. This movie is extracted from Supplementary Movie 1.

Supplementary Movie 3 Polarized optical microscope image of two tactoids merging. Two tactoids with three periodic bands fuse to give a new tactoid with five bands. This movie is extracted from Supplementary Movie 1.

Supplementary Movie 4 Polarized optical microscope image of several tactoids fusing. This movie is extracted from Supplementary Movie 1.

Supplementary Movie 5 Polarized optical microscope image of two tactoids fusing to leave a defect at their interface. This movie is extracted from Supplementary Movie 1.

## References

[b1] Habibi Y., Lucia L. A. & Rojas O. J. Cellulose nanocrystals: chemistry, self-assembly, and applications. Chem. Rev. 110, 3479–3500 (2010). 20201500 10.1021/cr900339w

[b2] Hamad W. Y. in ACS Symposium Series 1067 (eds Zhu, J. Y., Zhang, X. & Pan, X. J.) 301-321 (American Chemical Society (2011).

[b3] Moon R. J., Martini A., Nairn J., Simonsen J. & Youngblood J. Cellulose nanomaterials review: structure, properties and nanocomposites. Chem. Soc. Rev. 40, 3941–3994 (2011). 21566801 10.1039/c0cs00108b

[b4] Klemm D. . Nanocelluloses: a new family of nature-based materials. Angew. Chem. Int. Ed. 50, 5438–5466 (2011). 10.1002/anie.201001273 21598362

[b5] Lagerwall J. P. F. . Cellulose nanocrystal-based materials: from liquid crystal self-assembly and glass formation to multifunctional thin films. NPG Asia Mater. 6, e80 (2014).

[b6] Marchessault R. H., Morehead F. F. & Walter N. M. Liquid crystal systems from fibrillar polysaccharides. Nature 184, 632–633 (1959).

[b7] Revol J.-F., Bradford H., Giasson J., Marchessault R. H. & Gray D. G. Helicoidal self-ordering of cellulose microfibrils in aqueous suspension. Int. J. Biol. Macromol. 14, 170–172 (1992). 1390450 10.1016/s0141-8130(05)80008-x

[b8] Revol J.-F., Godbout L. & Gray D. G. Solid self-assembled films of cellulose with chiral nematic order and optically variable properties. J. Pulp Pap. Sci. 24, 146–149 (1998).

[b9] Roman M. & Gray D. G. Parabolic focal conics in self-assembled solid films of cellulose nanocrystals. Langmuir 21, 5555–5561 (2005). 15924489 10.1021/la046797f

[b10] Bouligand Y. Twisted fibrous arrangements in biological materials and cholesteric mesophases. Tissue Cell 4, 189–217 (1972). 4600349 10.1016/s0040-8166(72)80042-9

[b11] Sharma V., Crne M., Park J. O. & Srinivasarao M. Structural origin of circularly polarized iridescence in jeweled beetles. Science 325, 449–451 (2009). 19628862 10.1126/science.1172051

[b12] Dong X. M., Kimura T., Revol J.-F. & Gray D. G. Effects of ionic strength on the isotropic-chiral nematic phase transition of suspensions of cellulose crystallites. Langmuir 12, 2076–2082 (1996).

[b13] Shopsowitz K. E., Qi H., Hamad W. Y. & MacLachlan M. J. Free-standing mesoporous silica films with tunable chiral nematic structures. Nature 468, 422–425 (2010). 21085176 10.1038/nature09540

[b14] Hoeger I., Rojas O. J., Efimenko K., Velev O. D. & Kelley S. S. Ultrathin film coatings of aligned cellulose nanocrystals from a convective-shear assembly system and their surface mechanical properties. Soft Matter 7, 1957–1967 (2011).

[b15] Zhang Y. P. . in. Proceedings of SPIE 9172, Nanostructured Thin Films VII 91720R (2014).

[b16] Kelly J. A. . Responsive photonic hydrogels based on nanocrystalline cellulose. Angew. Chem. Int. Ed. 52, 8912–8916 (2013). 10.1002/anie.201302687 23881841

[b17] Orts W. J., Godbout L., Marchessault R. H. & Revol J.-F. Enhanced ordering of liquid crystalline suspensions of cellulose microfibrils: A Small Angle Neutron Scattering Study. Macromolecules 31, 5717–5725 (1998).

[b18] Araki J. & Kuga S. Effect of trace electrolyte on liquid crystal type of cellulose microcrystals. Langmuir 17, 4493–4496 (2001).

[b19] Hanley S. J., Revol J.-F., Godbout L. & Gray D. G. Atomic force microscopy and transmission electron microscopy of cellulose from *Micrasterias denticulata*; evidence for a chiral helical microfibril twist. Cellulose 4, 209–220 (1997).

[b20] Usov I. . Understanding nanocellulose chirality and structure-properties relationship at the single fibril level. Nat. Commun. 6, 7564 (2015). 26108282 10.1038/ncomms8564 PMC4491835

[b21] Elazzouzi-Hafraoui S. . The shape and size distribution of crystalline nanoparticles prepared by acid hydrolysis of native cellulose. Biomacromolecules 9, 57–65 (2008). 18052127 10.1021/bm700769p

[b22] Schütz C. . Rod packing in chiral nematic cellulose nanocrystal dispersions studied by small-angle x-ray scattering and laser diffraction. Langmuir 31, 6507–6513 (2015). 26020691 10.1021/acs.langmuir.5b00924

[b23] Sonin A. S. Inorganic lyotropic liquid crystals. J. Mater. Chem. 8, 2557–2574 (1998).

[b24] Bawden F. C., Pirie N. W., Bernal J. D. & Fankuchen I. Liquid crystalline substances from virus-infected plants. Nature 138, 1051–1052 (1936).

[b25] Robinson C. Liquid-crystalline structures in solutions of a polypeptide. Trans. Faraday Soc. 52, 571–592 (1956).

[b26] Revol J.-F. . Chiral nematic suspensions of cellulose crystallites; phase separation and magnetic field orientation. Liq. Cryst. 16, 127–134 (1994).

[b27] Dumanli A. G. . Controlled, bio-inspired self-assembly of cellulose-based chiral reflectors. Adv. Opt. Mater. 2, 646–650 (2014). 26229742 10.1002/adom.201400112 PMC4515966

[b28] Watson J. H. L., Heller W. & Wojtowicz W. Comparative electron and light microscopic investigations of tactoid structures in V_2_O_5_-sols. Science 109, 274–278 (1949). 17775051 10.1126/science.109.2829.274

[b29] Kaznacheev A. V., Bogdanov M. M. & Taraskin S. A. The nature of prolate shape of tactoids in lyotropic inorganic liquid crystals. J. Exp. Theor. Phys. 95, 57–63 (2002).

[b30] Prinsen P. & van der Schoot P. Shape and director-field transformation of tactoids. Phys. Rev. E 68, 021701 (2003). 10.1103/PhysRevE.68.021701 14524987

[b31] Park J. H. . Macroscopic control of helix orientation in films dried from cholesteric liquid-crystalline cellulose nanocrystal suspensions. ChemPhysChem 15, 1477–1484 (2014). 24677344 10.1002/cphc.201400062

[b32] Chen W. & Gray D. G. Interfacial tension between isotropic and anisotropic phases of a suspension of rodlike particles. Langmuir 18, 633–637 (2002).

[b33] Gray D. G. Iridescent films from cellulose nanocrystals: chiral nematic or smectic multi-lamellar structure? J-FOR 3, 6–8 (2013).

[b34] Majoinen J., Kontturi E., Ikkala O. & Gray D. G. SEM imaging of chiral nematic films cast from cellulose nanocrystal suspensions. Cellulose 19, 1599–1605 (2012).

[b35] Hirai A., Inui O., Horii F. & Tsuji M. Phase separation behavior in aqueous suspensions of bacterial cellulose nanocrystals prepared by sulfuric acid treatment. Langmuir 25, 497–502 (2009). 19055323 10.1021/la802947m

[b36] Gray D. G. & Mu X. Chiral nematic structure of cellulose nanocrystal suspensions and films; polarized light and atomic force microscopy. Materials 8, 7873–7888 (2015). 10.3390/ma8115427 PMC5458898 28793684

