# Supplementary material for: Structure and transformation of tactoids in cellulose nanocrystal suspensions
Source: Nat Commun. 2016 May 4;7:11515. doi: 10.1038/ncomms11515 (PMC4857480; doi:10.1038/ncomms11515)
Supplement: Supplementary Information — Supplementary Figures 1-13, Supplementary Tables 1-2, Supplementary Methods and Supplementary Reference [file ncomms11515-s1.pdf]

Supplementary Materials

Supplementary Figures

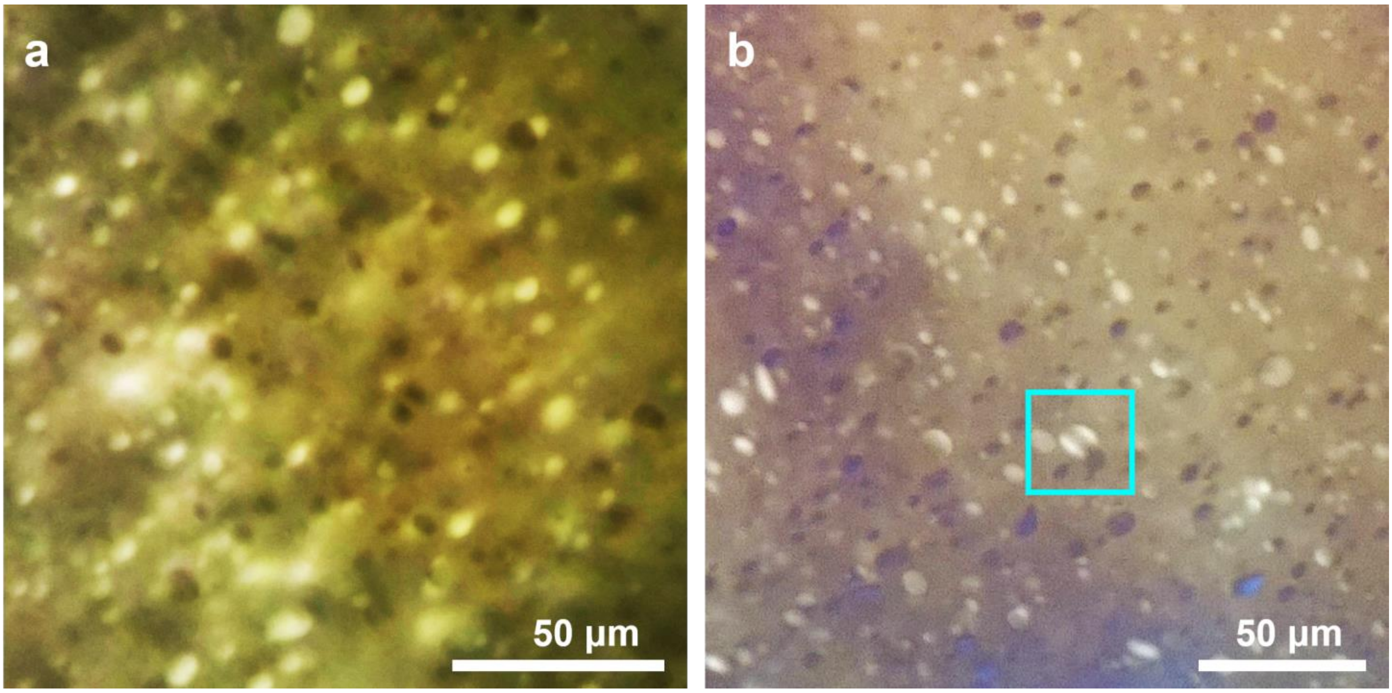

Supplementary Figure 1. POM images for newborn CNC tactoids. (**a**) POM image of newborn "baby" tactoids in 4 wt% CNC suspension after sonication. (**b**) POM image showing a tactoid with one birefringent band, which is indicated by a rectangle.

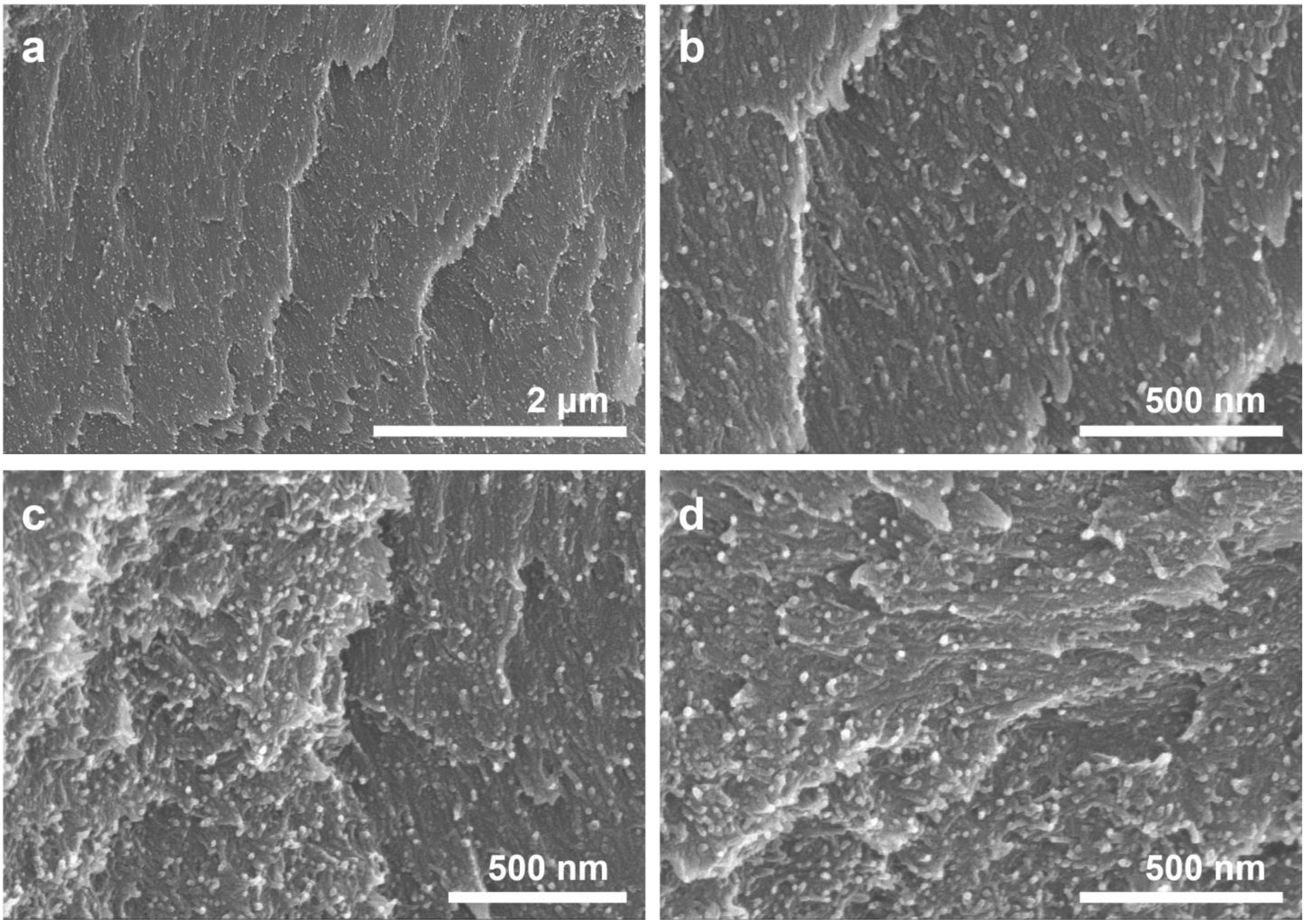

Supplementary Figure 2. Additional SEM images for the tactoid in Figure 2. (**a**)-(**b**) SEM images showing the nematic arrangements of CNCs inside this "baby" tactoid. (**c**) Magnified view for its left edge. (**d**) Magnified view for its bottom edge.

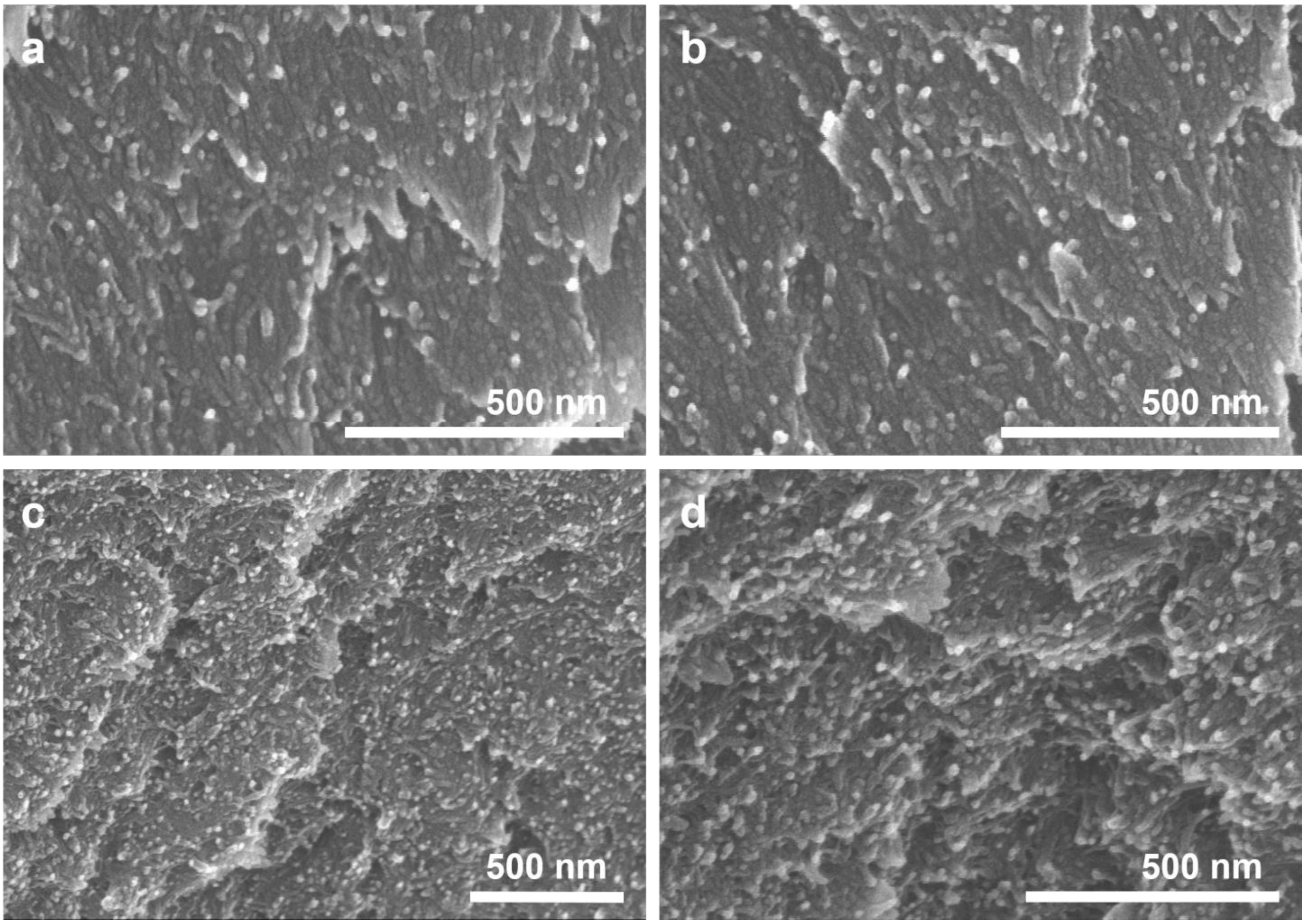

Supplementary Figure 3. Inside and outside the tactoid in Figure 2. (**a**)-(**b**) SEM images showing the parallel arranged CNCs inside the tactoid. (**c**)-(**d**) The randomly arranged CNCs in nearby isotropic regions outside the tactoid.

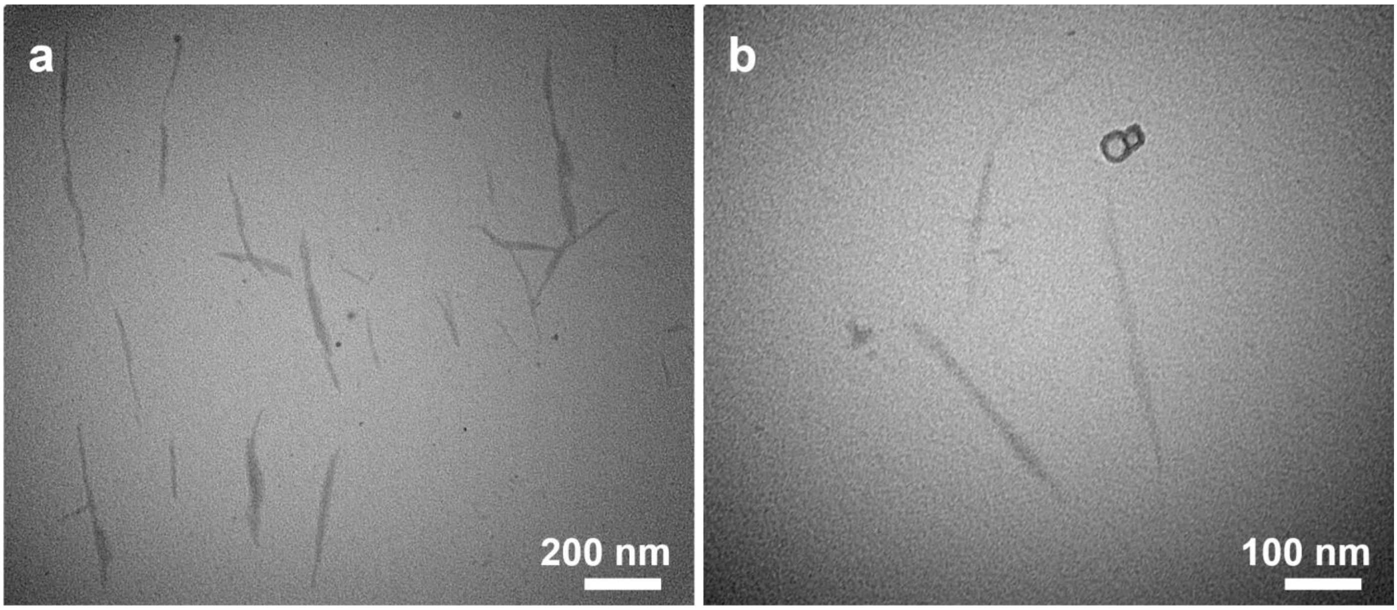

Supplementary Figure 4. TEM images of cellulose nanocrystals used in these studies. (**a**) Cellulose nanocrystals dispersed from water. (**b**) Cellulose nanocrystals dispersed from water, shown at higher magnification.

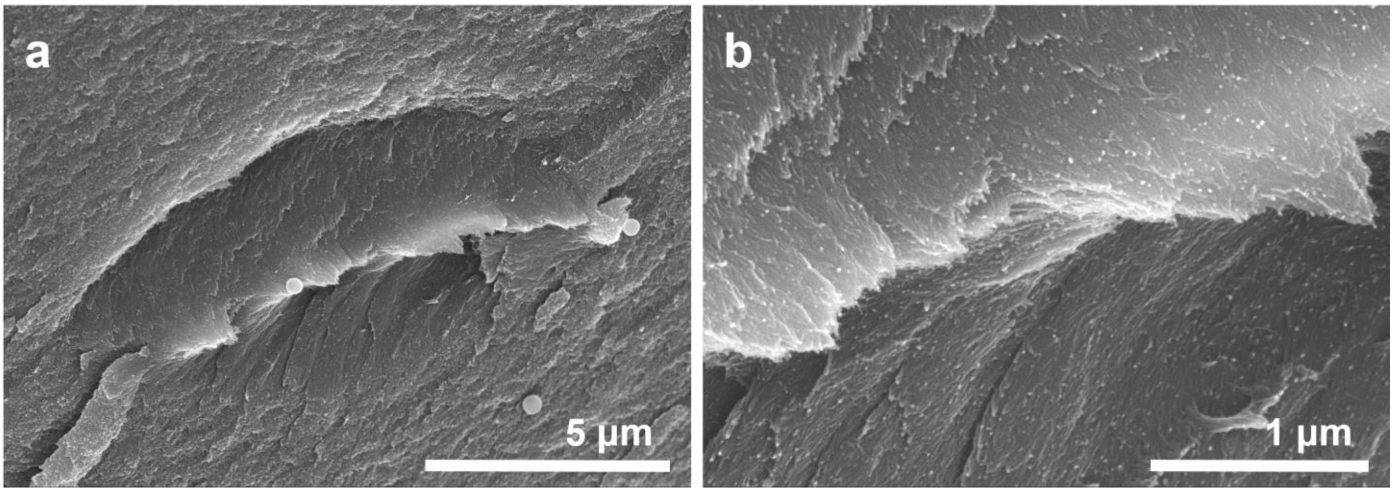

Supplementary Figure 5. A tactoid with one band. (**a**) SEM image of a tactoid with only one periodic band. (**b**) Expanded view for its band.

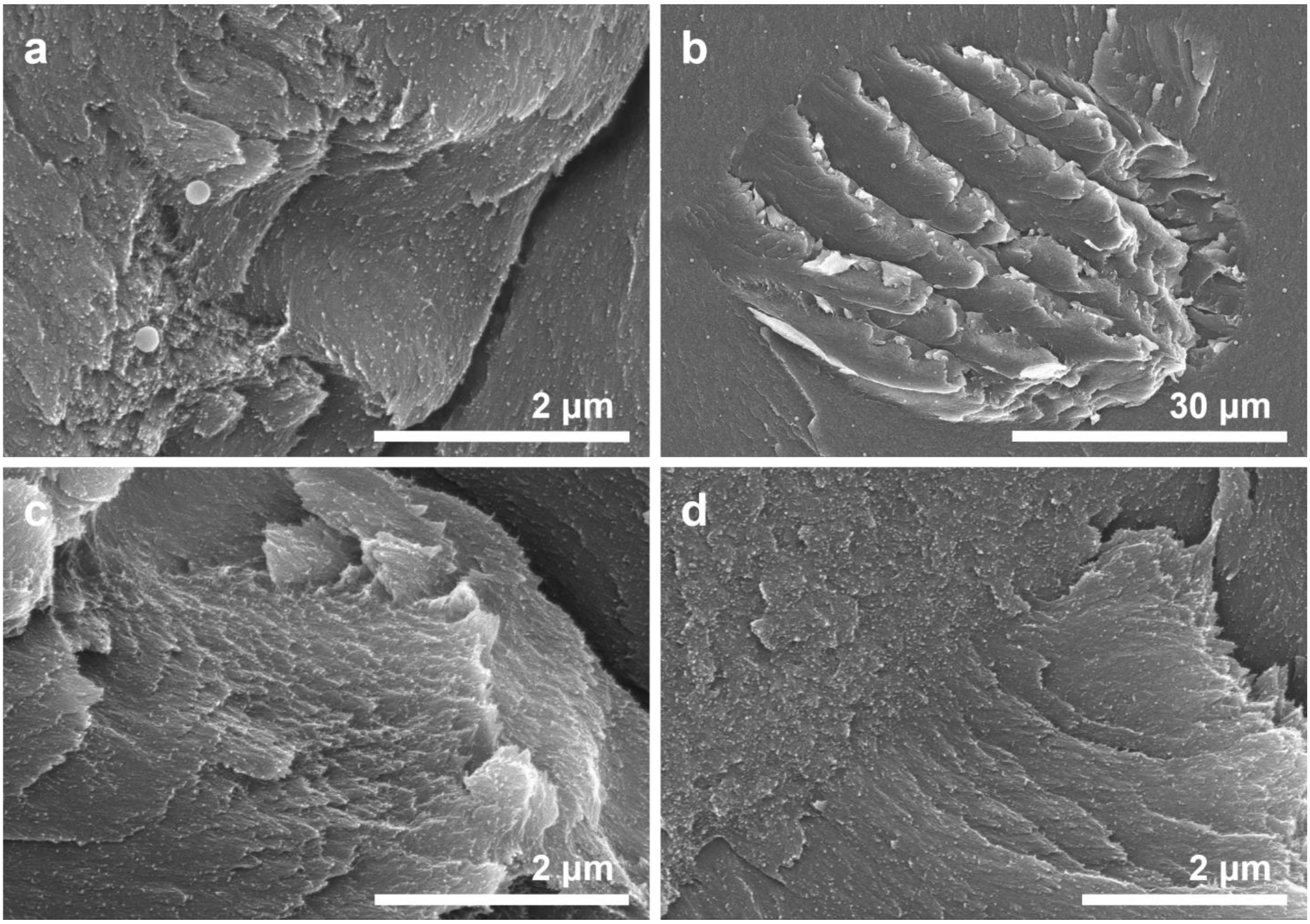

Supplementary Figure 6. Stepped geometry of the periodic bands. (**a**) SEM image of the same tactoid in **Figure 3c**, a cross-section of one of its periodic bands is shown here. SEM image (**b**) shows a similar tactoid with 6 periodic bands, a cross-section of which is shown in (**c**), while an edge of this tactoid is shown in (**d**).

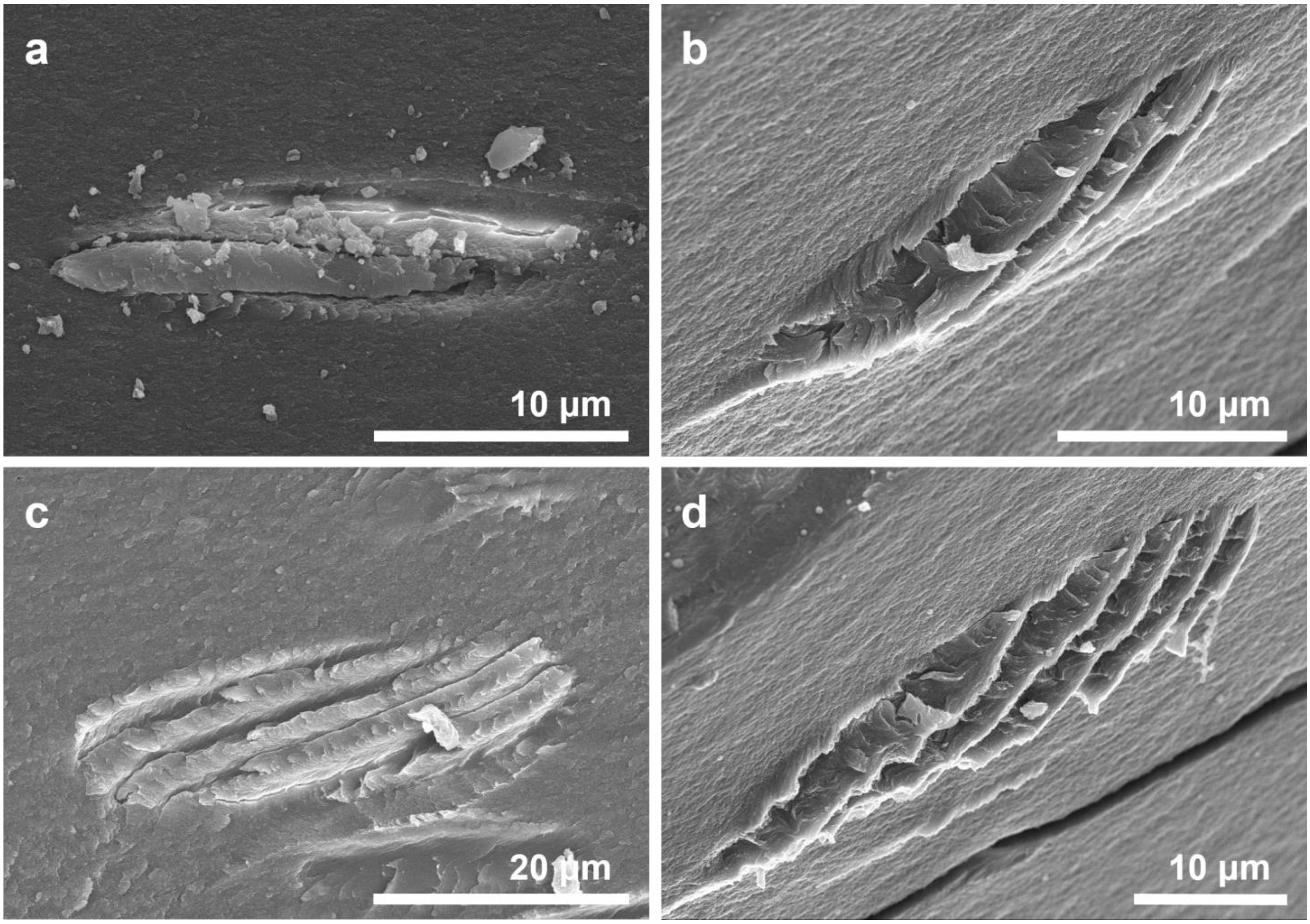

Supplementary Figure 7. SEM images of tactoids with thick sheets. Each of these sheets contains a half helical pitch.

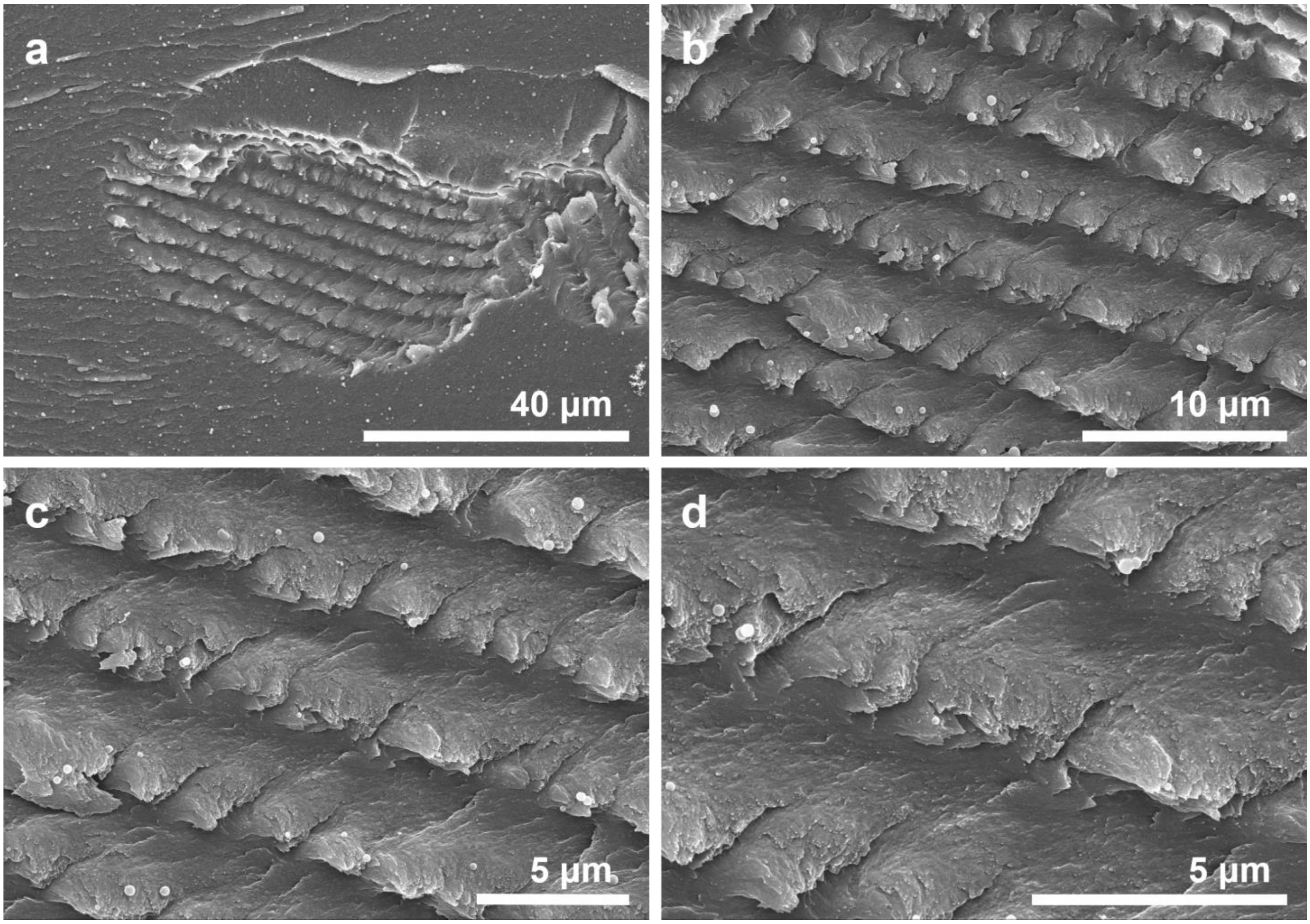

Supplementary Figure 8. Additional SEM images of the tactoid in Figure 3e.

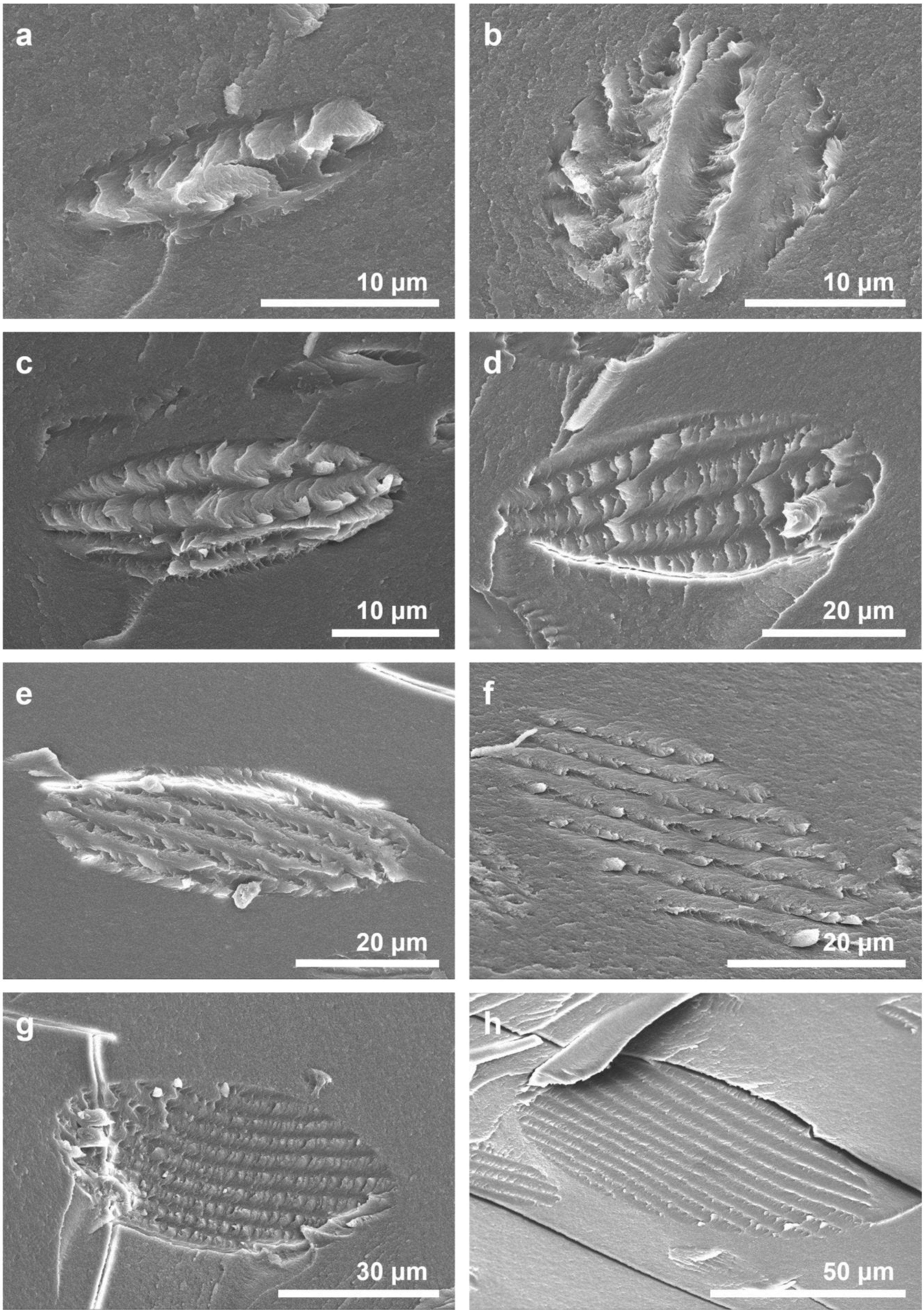

Supplementary Figure 9. Tactoids with different numbers of bands. SEM images of tactoids with (**a**) 1, (**b**) 2, (**c**) 3, (**d**) 4, (**e**) 5, (**f**) 7, (**g**) 9, and (**h**) 12 periodic bands.

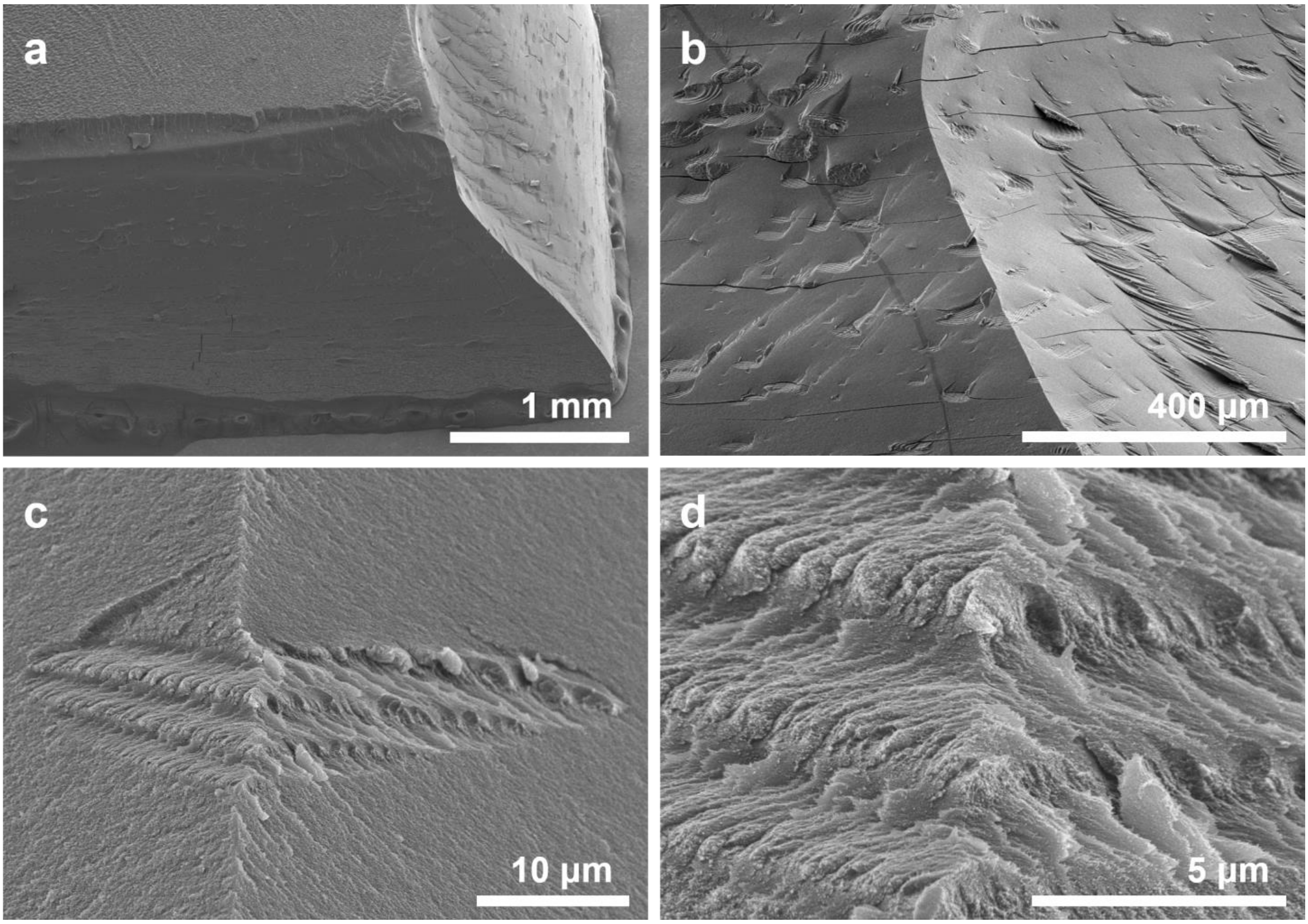

Supplementary Figure 10. Tactoids at a right angled edge. (**a**) SEM image for a piece of dried gel with a right-angled cross-section, (**b**) shows a tactoid in the center sitting at this cross-section, which is the same one as that in **Figure 4** and **Figure 5**. (**c**) is another tactoid at the same right-angled cross-section; its chiral nematic structure is shown with a higher magnification in (**d**).

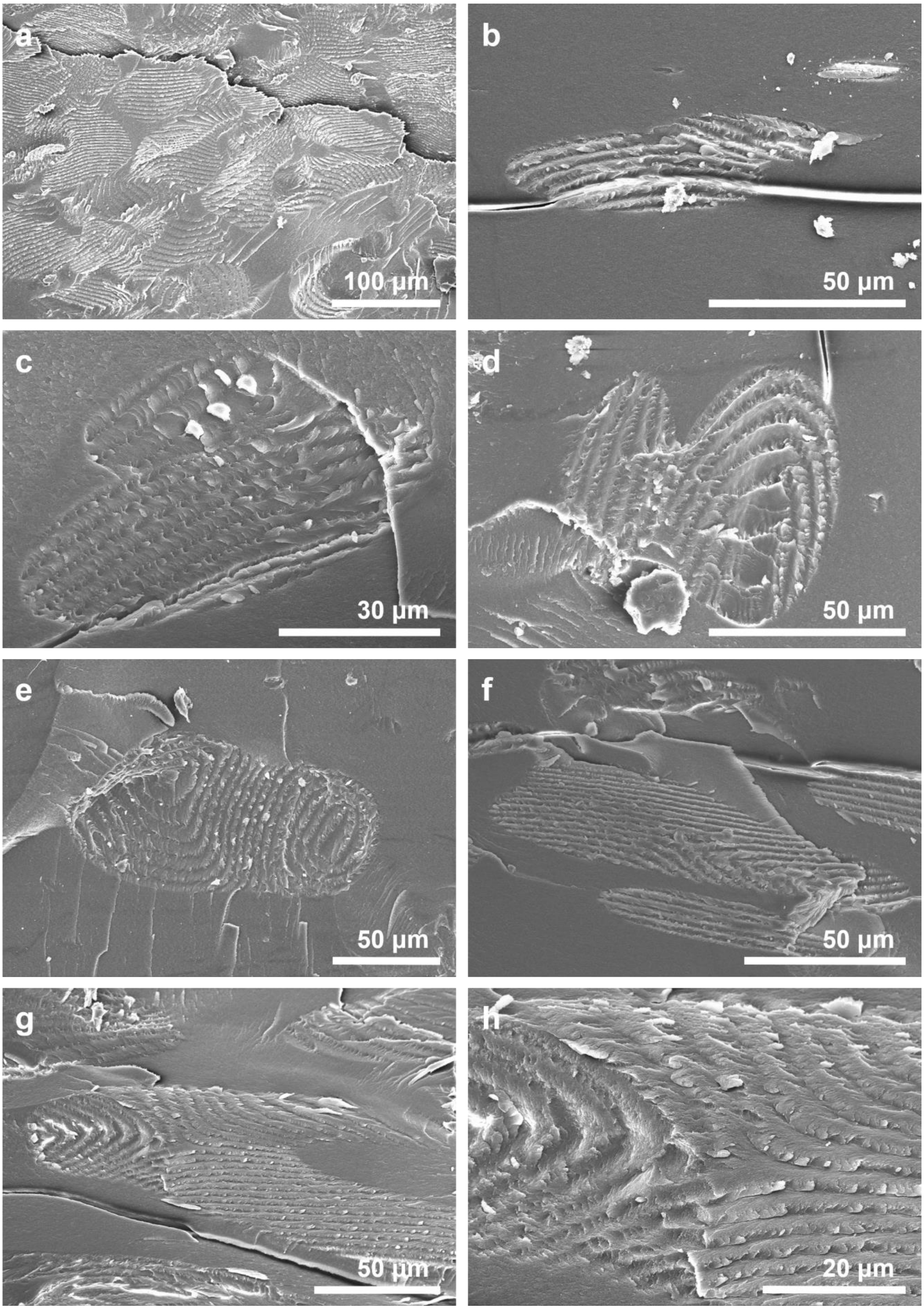

Supplementary Figure 11. Fusion of tactoids. (**a**) SEM image showing the aggregation of tactoids in a region near the bottom of a dried gel. (**b**)-(**d**) The collisions between tactoids. (**e**)-(**g**) Hybrid tactoids formed by fusions. (**h**) shows a magnified view of the fusion defect in the hybrid tactoid shown in (**g**).

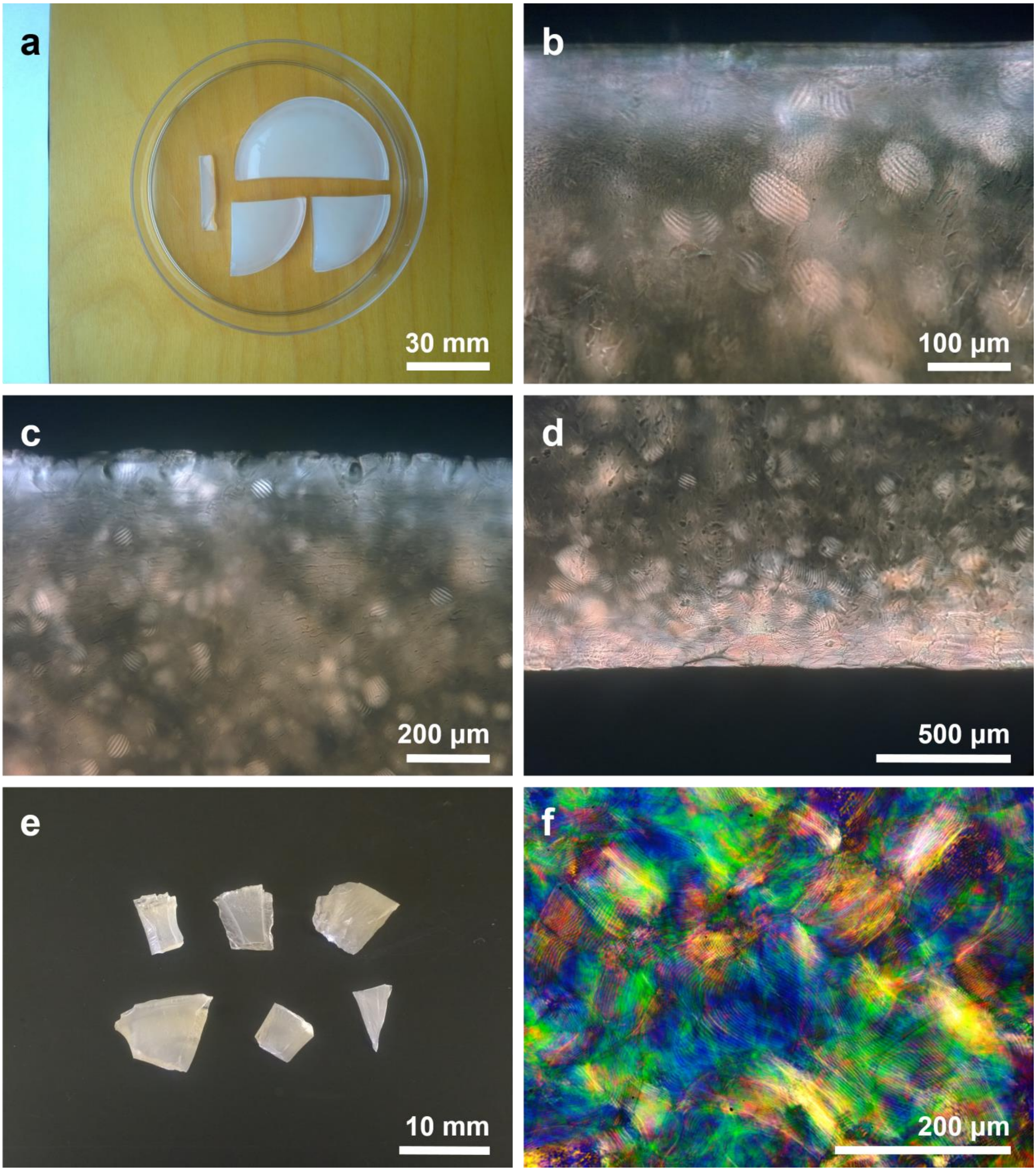

Supplementary Figure 12. Additional images for this work. (**a**) shows pieces of a freshly sliced hydrogel. (**b**)-(**d**) are POM images for the section in (**a**), which show tactoids captured in the hydrogel, and the aggregation of tactoids at the bottom of the CNC suspension. (**e**) shows some pieces of dried gels obtained with a hammer. (**f**) is a POM image showing the fingerprints of a dried CNC film.

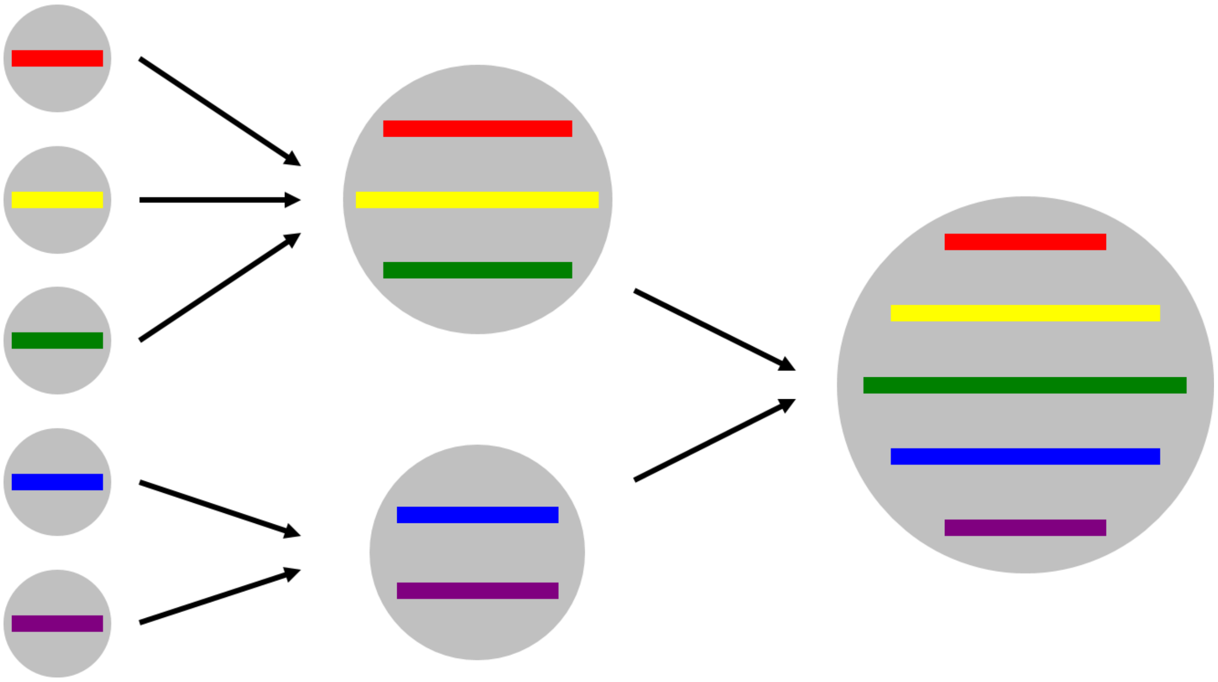

Supplementary Figure 13. Fusion process of tactoid. A cartoon depiction for an idealized fusion process from five one-band small tactoids to one five-band big tactoid. We hypothesize that different periodic bands in a big tactoid would be collected from different small tactoids through fusion processes.

Supplementary Table 1. Mass decrease and concentration increase of a CNC-PAAm precursor mixture during evaporation.[a]

Time	0 h	0.5	1 h	2 h	3 h	4 h	5 h
Mass (g)	10.7397	10.6523	10.5824	10.4382	10.2980	10.1507	9.9993
Conc. (wt.%)	4.00	4.04	4.07	4.13	4.19	4.26	4.33
6 h	7 h 5 min	8 h	9 h	10 h	10 h 40 min	11 h 30 min	12 h
9.8501	9.6832	9.5496	9.3997	9.2383	9.1345	8.9937	8.9137
4.41	4.49	4.56	4.64	4.74	4.80	4.88	4.93

[a] In order to determine the approximate concentration of CNCs during the evaporation, we undertook an experiment identical to the ones reported in the paper, weighing the solution as a function of time. Initially, a 4 wt.% CNC suspension (9.9184 g) was mixed with 1.00 g acrylamide, 100 mg cross-linker and 5 mg photo-initiator in a vial. 10.7397 g of this mixture (97.43% of 11.0234 g) was transferred into a 60 mm diameter Petri dish; this mixture contains 0.3865 g CNCs and 9.2766 g water in theory. Based on this information, the concentrations of CNCs at different time points are calculated. The mass of this CNC-PAAm precursor mixture decreased linearly with time, $m = 10.744 - 0.1508t$, $R^2 = 0.9997$ (m in grams, t in hours). The wt% CNCs are based on the wt% CNCs per unit water, neglecting the added PAAm, cross-linker, and initiator, and we assumed that only water evaporates from the mixture over this time period. Also, the experiment was performed on a different day than the ones for the images, but we believe that the humidity was similar (~70%) and the approximate concentrations are correct.

Supplementary Table 2. Average spacing between adjacent periodic bands in 29 CNC tactoids having different numbers of bands.[a]

Bands	2	3	3	3	3	4	4	4	4
Spacing (μm)	6.020	4.805	3.664	3.222	3.298	5.405	5.668	2.579	5.122
4	4	4	5	5	6	6	6	6	7
4.199	2.605	2.498	3.590	2.687	2.881	3.729	3.991	2.934	2.322
7	7	7	8	8	9	9	9	10	12
4.750	3.757	2.371	1.966	3.579	3.991	3.071	2.372	1.978	2.871

[a] Distances were determined by measuring the total edge-to-edge distance (SEM) and dividing by the number of bands. The spacing between adjacent bands corresponds to one half helical pitch.

Supplementary Methods

Acrylamide (Aldrich, 98%), N,N'-methylenebisacrylamide (Aldrich, 99%), 2,2-diethoxyacetophenone (Acros, 98%), and 2-hydroxy-4'-(2-hydroxyethoxy)-2-methylpropiophenone (Aldrich, 98%) were used without further purification. CNC suspensions (4 wt.% in water, σ = 2.19 mS·cm^{-1}, pH = 2.44) were provided by CelluForce. The CNCs were obtained from hydrolysis of wood pulp in sulfuric acid using the literature procedure.[1] TEM of these crystals gave dimensions of 19±9 nm by 245±135 nm (based on measuring 98 crystallites).

Optical microscopy was performed with an Olympus BX41 microscope. Scanning electron microscopy (SEM) experiments were conducted on a Hitachi S4700 electron microscope at an acceleration voltage of 10 kV. Samples were sputter-coated with 8 nm of platinum-palladium (80:20) alloy before imaging. Transmission electron microscopy (TEM) images were obtained with a Hitachi H7600 electron microscope at an acceleration voltage of 62 kV. Samples for TEM measurements were prepared by diluting an aqueous suspension of CNCs to ~0.002 wt.%, sonicating the dilute solution for 3 h, then drop-casting the suspension onto carbon-coated copper grids. The thickness of hydrogels and dried gels was measured with a MARATHON Electronic Digital Micrometer. The diameter of dried gels was measured with a ruler. A Durasonix 3 Litre Ultrasonic Cleaner (Power: 120 W; Frequency: 40 kHz) was used in this work for sonication treatments of CNC suspensions.

Supplementary Reference

[1] Hamad, W. Y. & Hu, T. Q. Structure–process–yield interrelations in nanocrystalline cellulose extraction. *Can. J. Chem. Eng.* **88,** 392−402 (2010).
